# Antimicrobial susceptibility patterns of urinary tract infections causing bacterial isolates and associated risk factors among HIV patients in Tigray, Northern Ethiopia

**DOI:** 10.1186/s12866-024-03297-2

**Published:** 2024-04-27

**Authors:** Tsgabu Kahsay, Gebrecherkos Teame Gebrehiwot, Gebreselema Gebreyohannes, Mulugeta Tilahun, Ataklti Gessese, Amlisha Kahsay

**Affiliations:** 1 Department of Microbiology and Immunology, Dr. Tewelde Legesse College of Health Sciences, Mekelle, Tigray, Ethiopia; 2 Department of Biomedical Research and Technology Transfer, Tigray Health Research Institute, Mekelle, Tigray, Ethiopia; 3 Department of Biological and Chemical Engineering, Mekelle Institute Technology, Mekelle University, Mekelle, Tigray, Ethiopia; 4 Department of Medical Microbiology and Immunology, College of Health Sciences, Mekelle University, Mekelle, Tigray, Ethiopia

**Keywords:** Antimicrobial susceptibility pattern, Bacterial isolates, HIV patients, UTIs

## Abstract

**Background:**

Urinary tract infections, a prevalent global infectious disease, are clinical issues not well studied in HIV-positive individuals. UTIs have become a global drug resistance issue, but the prevalence and antibiotic susceptibility patterns of UTI-causing bacteria among HIV patients in Tigray, Ethiopia, are poorly understood. This study aims to identify the prevalence of UTI-causing bacteria, their antibiotic susceptibility patterns, and associated risk factors in HIV patients attending ART clinics at Mekelle General Hospital and Ayder Comprehensive Specialized Hospital in Tigray, Northern Ethiopia.

**Method:**

Clean-catch midstream urine samples (10–15 mL) were collected from HIV patients who are attending ART clinics at Mekelle General Hospital and Ayder Comprehensive Specialized Hospital. Samples were analyzed based on standard microbiological protocols using cysteine-lactose electrolyte deficient (CLED) agar. Pure colonies of bacterial isolates were obtained by sub-culturing into Mac-Conkey, Manitol Salt agar and blood agar plates. The bacterial isolates were then identified using macroscopic, microscopic, biochemical, and Gram staining methods. Gram-negative bacteria were identified using biochemical tests like triple sugar iron agar, Simon’s citrate agar, lysine iron agar, urea, motility test, and indol test, whereas Gram-positive isolates were identified using catalase and coagulase tests. The Kirby-Bauer disk diffusion technique was used to analyze the antimicrobial susceptibility pattern of bacterial isolates. Data was analyzed using SPSS version 25.0.

**Results:**

Among the 224 patients, 28 (12.5%) of them had been infected by UTIs-causing bacteria. *E. coli* was the dominant bacterium (16 (57%)) followed by *K. pneumoniae* (4 (14%)), and *S. aureu*s (3 (11%)). Of the total bacterial isolates, 22 (78.6%) of them developed multi-drug resistance. All Gram-positive (100%) and 75% of Gram-negative bacterial isolates were found to be resistant to two or more drugs. Patients with a history of UTIs, and with CD_4_ count < 200 cells/ mm^3^, were more likely to have significant bacteriuria. Compared to male patients, female patients were more affected by the UTIs-causing bacteria. More than 93% of the UTIs-causing bacterial isolates were susceptible to nitrofurantoin, ceftriaxone, ciprofloxacin, and gentamycin; whereas they are highly resistant to ampicillin (96%), cotrimoxazole (82%) and tetracycline (71%).

**Conclusions:**

Most of the bacterial isolates were highly resistant to ampicillin, cotrimoxazole, and tetracycline. Female patients were more affected by the UTIs causing bacteria. The highest prevalence (12.5%) of UTIs in HIV patients needs special attention for better management and monitoring. Previous UTI history and immune suppression are predictors of UTIs, highlighting the need for intervention measures involving molecular studies to identify resistant bacteria genes and promote patient immune reconstitution.

**Supplementary Information:**

The online version contains supplementary material available at 10.1186/s12866-024-03297-2.

## Background

Urinary tract infections (UTIs) are significant quantities of microbial pathogens in the urinary tract, including urethra, bladder, ureters, kidneys, or prostate [1, 2]. UTIs are among the most common infectious diseases worldwide but are significantly understudied [3]. UTIs are one of the most common bacterial infections globally, with an estimated annual incidence of more than 150 million cases worldwide and costing the global economy more than 6 billion US dollars [4, 5].

Urinary tract infections (UTIs) are prevalent clinical issues involving bacterial invasion and multiplication in the urinary tract system’s organs, accounting for 1–6% of medical referrals and affecting urinary tract, bladder, and kidney infections [6, 7]. It is the second most prevalent bacterial infection, affecting people of all ages all over the world [8]. Urinary tract infections (UTIs) remain to be one of the most common infectious diseases diagnosed in developing countries [9]. The burden of recurrent UTIs has both personal (social and psychological effects) and societal aspects (clinical and economic burden of the illness) which harm the quality of life [10].

As of 2022, over 80% of the 39 million HIV-infected individuals worldwide, including 1.8 million children, are from the WHO Africa region [11]. Despite increasing coverage, the UNAIDS goal of 95% coverage by 2025 remains unrealistic [12]. Investing in research, education, awareness campaigns, and access to ART, along with comprehensive HIV prevention techniques like pre-exposure prophylaxis, condoms, and safe sexual practices, is crucial for combating HIV [11].

People living with human immunodeficiency virus (HIV) are more likely to develop urinary tract infections (UTIs) due to the suppression of their immunity [13]. Asymptomatic UTIs among HIV patients can progress to symptomatic ones characterized by mild irritation, bacteremia, sepsis, and death [13, 14]. UTIs among HIV patients can bring numerous health consequences, including acute and chronic kidney diseases, infertility, cancer, sepsis, and neurologic complication which could lead to urinary stasis [15]. HIV patients may face significant financial burden due to UTIs recurrence, expensive antimicrobials, extended hospital stays, adverse drug effects, and unsatisfactory therapeutic options, which may lead to further complications [4, 16].

Studies indicate a global increase in UTI prevalence in HIV/AIDS patients, ranging from 6.3 to 77.5% [4, 17, 18]. UTIs may lead to hospitalization of HIV-infected patients [13, 18]. Bacteria that cause UTIs among HIV patients include *Escherichia coli, Enterococcus species, Pseudomonas aeruginosa, Proteus species, Klebsiella species*, and *Staphylococcus aureus* [19, 20] *.*

The emergence of antibiotic resistance is particularly enormous in developing countries [20]. This is because of having low-quality laboratory facilities to isolate pathogens and determine their antimicrobial susceptibility pattern and due to the misuse of antimicrobials [20, 21]. Resistant bacteria are more difficult to treat even at higher doses [22, 23]. Ethiopian studies reveal a concerning rise in urinary tract infections (UTIs) due to the high drug resistance of isolated uropathogens [19, 24]. This has a significant impact on bacterial infection management, resulting in higher mortality, morbidity, and treatment costs [25, 26].

Studies from various corners of the globe have identified many risk factors associated with UTIs among patients with HIV. Patients with CD4^+^ cell count < 200 cells/mm^3^, HIV-positive females, and patients with conditions that may obstruct urine flow like enlarged prostate, congenital urinary tract abnormalities, and inflammation were cited as more likely to experience UTIs [27, 28]. Previous history of UTIs, current symptoms of UTIs, and previous history of catheterization were risk factors associated with UTIs cited among studies from Ethiopia [29].

Ethiopia has limited research on the extent of UTIs causing bacterial isolates and antimicrobial susceptibility patterns in HIV-1 infected patients, with no published study in the selected study area. This study investigated the prevalence of UTIs caused by bacteriuria isolates, antimicrobial susceptibility patterns, and associated risk factors among HIV-infected patients in Tigray, Ethiopia, attending ART clinics.

## Materials and methods

### Study area

The study was conducted at Ayder Comprehensive Specialized Hospital (ACSH) and Mekelle General Hospital (MGH), Mekelle, northern Ethiopia. Mekelle is located 783 km to the north of Addis Ababa at an altitude and longitude of 13,029’N 39,028’E, respectively with an elevation of 2084 m above sea level. The city has a total population of 586,897 [30]. ACSH, governed by Mekelle University, is the largest hospital in the region with 450 beds, while MGH, governed by the Regional Health Bureau, has 166 beds.

These hospitals serve patients who come from all parts of the region (which comprises about 7 million people) and from Afar and Amhara Regional States. ACSH and MGH provide ART services for up to 1,550 and 4,495 patients, respectively.

### Study design and period

Between February and June 2021, a cross-sectional study was conducted at health facilities.

### Sampling technique and sample size determination

The sample size was determined using single proportion formula, N_1_ = Z^2^α/2 P (1- P)/d^2^, N1 was the initial sample size, with a 95% confidence level, an estimated prevalence of bacterial UTIs among HIV patients 15.8% (*p* = 0.158) [31], and a precision of 3% after considering the 10% non-response rate, N1 = 224 and, using the formula for proportionate allocation the sample size for each health facility (N2) was 57 and 167 in ACSH and MGH, respectively.

### Source of study population

The study included all HIV-infected individuals, regardless of age, who visited ACSH and MGH’s ART clinics.

### Recruitment criteria

The inclusion criteria for UTIs included symptomatic or asymptomatic presentations, being on ART or pre-ART, and having a history of UTIs. Patients who took antibiotics, including cotrimoxazole prophylaxis, but didn’t give consent two weeks before data collection and didn’t take their ART medications were excluded from the study.

### Study variables

The prevalence of UTIs causing bacterial isolates and antibiotic susceptibility patterns were considered primary and secondary outcome variables. Socio-demographic characteristics including (age, gender, residence, marital status, occupation, and level of education) and Clinical characteristics briefly including a history of UTIs, symptoms of UTIs, previous history of catheterization, CD4^+^ cell count, and viral load level were predictor variables.

### Data collection procedure

The study collected prospective data on the socio-demographic characteristics of patients using a structured questionnaire designed specifically for this purpose. The study also collected clinical data, including clinical history, CD4 + cell count, and viral load levels, retrospectively using standardized checklists, before incorporating socio-demographic data and urine sample collection.

### Urine collection

An adequate explanation of how to collect the specimen was provided by trained professional personnel. Sterile, dry, wide-necked, and leak-proof containers were prepared for sample collection [32]. Containers were labeled with a unique sample number, date, and time of collection. Briefly, about 10 to 15 mL of clean-catch midstream urine sample was collected from each patient. The collected samples were delivered immediately to the College of Health Sciences Medical Microbiology Laboratory and processed within two hours [33].

### Identification of bacterial isolates

**Fig. 1:**
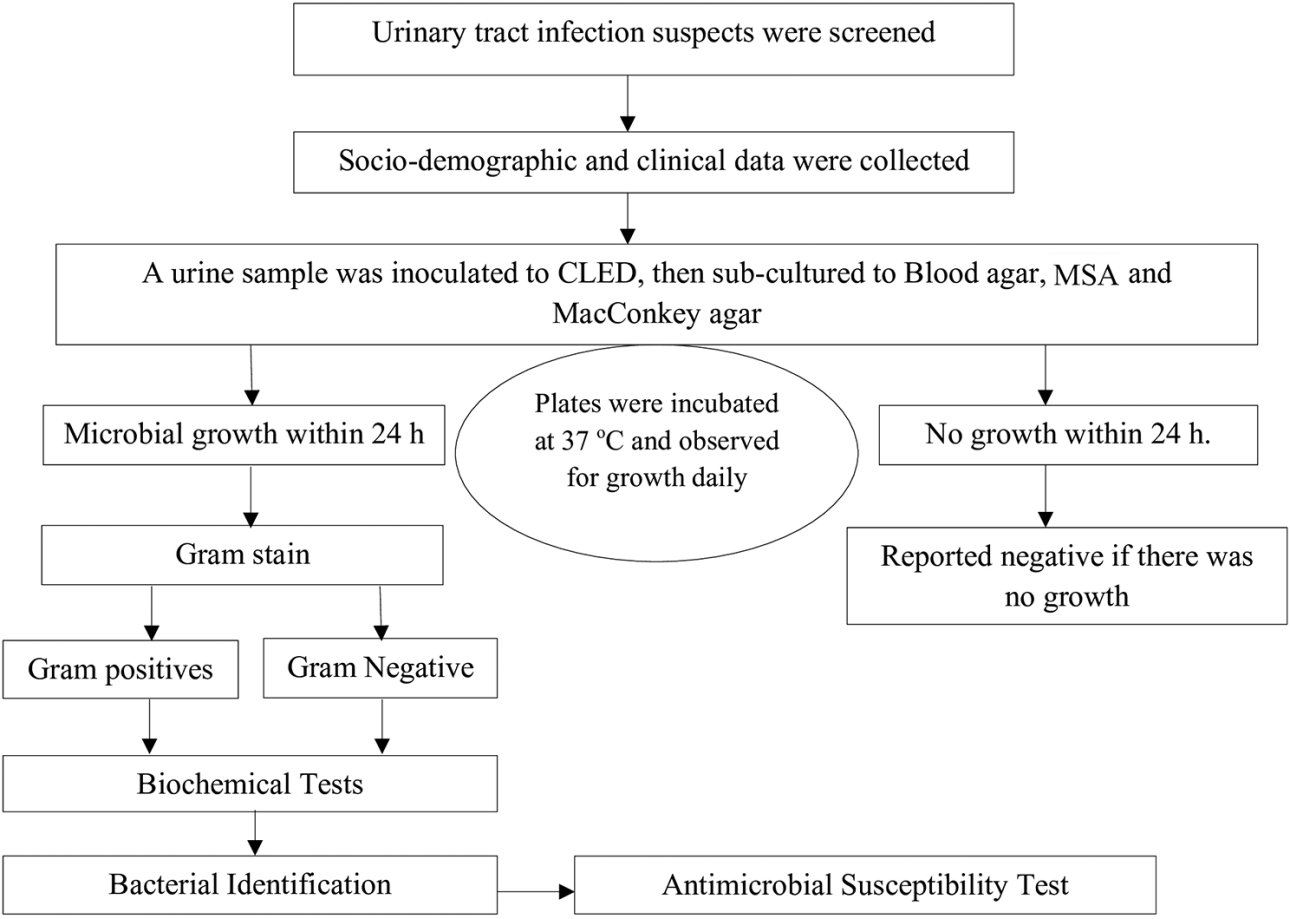
Schematic representation of the experiment workflow

Figure 1 shows the whole procedures summarized schematically. Briefly; 0.001 ml of well-mixed un-centrifuged urine was inoculated into cysteine-lactose electrolyte deficient (CLED) agar using a calibrated sterile wire loop (Oxoid, UK). It was aerobically incubated at 37 °C for 18–24 h and examined for bacterial growth. About 3–5 pure colonies from the CLED agar were sub-cultured into Mac-Conkey agar (Oxoid, UK), blood agar plates and Manitol salt agar (MSA) incubated at 37 °C for 18–24 h to differentiate and select our isolates of interest. If bacterial isolates were grown from the urine samples (≥ 10^5^ CFU/mL), it was considered significant. Bacterial isolates were identified using colony characteristics, Gram reaction, and biochemical tests. Gram-negative bacteria were identified using a series of biochemical tests such as triple sugar iron agar, Simon’s citrate agar, lysine iron agar, urea, motility test, and indol test. Whereas Gram-positive bacterial isolates were identified using catalase and coagulase tests [33–35].

### Antimicrobial susceptibility test

An antimicrobial susceptibility test was done using the Kirby-Bauer disk diffusion method. Different antibiotic discs (Oxoid Ltd, UK) such as ampicillin (10 µg), ciprofloxacin (5 µg), gentamicin (10 µg), cotrimoxazole (25 µg), chloramphenicol (30 µg), meropenem (10 µg), nitrofurantoin (300 µg), ceftriaxone (30 µg), clindamycin (15 µg), norfloxacin (10 µg), tetracycline (30 µg), penicillin (10 µg), erythromycin (15 µg), and cefoxitin (30 µg) were used. The antibiotics were chosen based on their availability and frequent prescription in the study area by adhering to the Standards for Clinical Laboratory International (SCLI).

Briefly, 3–5 pure similar colonies were picked using a sterile wire loop and mixed in 5 mL of normal saline until the turbidity of the suspension matched 0.5 McFarland standards. A portion of suspension was inoculated on the surface of the Mueller Hinton agar plate using a dry and sterile cotton swab. After 10 min, different antibiotic discs were placed on the media and incubated at 37 ^o^C for 24 h. After 24 h of incubation, the zone of inhibition (cm in diameter) around the discs was measured and interpreted as sensitive (S), intermediate (I), or resistant (R) based on the Clinical Laboratory Standard Institute (CLSI) guidelines of 2018 [36, 37].

### Data management, analysis, and interpretation

Data was analyzed using SPSS Statistical Software Ver. 25.0. Descriptive statistics were computed and results were described using tables and figures. To minimize potential confounders, covariates with *p* ≤ 0.25 in the bivariate analysis were made eligible in the multivariable regression model which was employed to determine the association of potential predictors with the respective outcome variables. Compression between subgroups was expressed as an odds ratio (AOR) with a 95% confidence interval (CI). *P* < 0.05 was used to declare statistical significance.

### Operational definitions

Urinary tract infection is the presence of pathogenic organisms within the urinary tract in a significant quantity (≥ 10^5^ CFU/mL) [13]. Asymptomatic UTIs are the presence of significant bacteria (≥ 10^5^ CFU/mL) in an individual’s urine without signs and symptoms of UTIs [1]. Symptomatic UTIs are characterized by a patient’s presence of fever, urgency, frequency, dysuria, or suprapubic tenderness [38]. Multi-Drug Resistant (MDR) is defined as when the isolated bacteria are resistant to two or more drugs from different classes [39]. Bacteriuria refers to the unusual presence of bacteria in midstream urine, with a colony count exceeding 10^5^ CFU/mL [40].

### Data quality assurance

To ensure the quality of socio-demographic and clinical data, a structured questionnaire was pretested. Data collectors (nurses) were trained for one day. The sterility of culture media was checked by incubating 5% of the culture media overnight at 35–37 °C without specimen inoculation. The performance of culture media was checked by litigating control strains. Standard strains of *E. coli* (ATCC 25,922) and *S. aureus* (ATCC 25,923) were obtained from the Ethiopian Public Health Institute laboratory (EPHI) to control the performance of culture media and antibiotic discs. Any physical changes like cracks, excess moisture, color, hemolysis, dehydration, and contamination were assessed and the expiry date was also checked. As per the recommendations of the International Clinical Laboratory Standard (CLSI), standard operating procedures (SOPs) were followed and applied throughout the analysis.

### Ethical considerations

Ethical clearance was obtained from the Institutional Research and Ethical Review Board (IERB) of the College of Health Sciences, Mekelle University with reference number (*MU-IRB1827/2021)*.All the methods were performed in accordance with relevant national, international and scientific guidelines and regulations. Besides, our study was carried out in accordance with the code of ethics of the world medical association (Declaration of Helsinki) for experiments in humans. After the objective of the study was explained, before collecting the data, informed consent and assent were collected from adult participants and minors’ guardians, respectively. Participants were informed of their right to withdraw from the study and were informed about the study’s benefits to their medications and the community at large. All information, samples and experimental results obtained were kept confidential thoroughly and used for the specified objectives only. Finally, the specimens were discarded following the infection prevention guide line.

## Results

### Socio-demographic, clinical data characteristics, and prevalence of UTIs among the study patients

Of the 224 HIV patients selected, 150/224 (67%) were females with a mean age of 40 (± 10.60) years (Table 1). The majority of the study patients were aged between 35 and 44 years. Among the study patients, 89 (39.7%) were married, 88 (39.3%) completed their primary school education, and 78 (34.8%) were daily laborers. Clinical data of the patients indicate that 20 (8.9%) of them had other chronic diseases, 25 (11.2%) with less than 200 CD4^+^ cells/mm^3^, 202 (90.2%) with no detectable level of current HIV viral load, 192 (85.7%) good level of ART adherence, 27(12.1%) with a previous history of catheterization, and 30 (13.4%) with a previous history of UTIs. On the other hand, 30 (13.4%) and 194 (86.6%) of the study patients were categorized as UTIs symptomatic and asymptomatic, respectively. Of the total 224 patients, 28 (12.5%) of the patients were infected by UTIs-causing bacteria. Compared to female patients 24/150 (16%), male patients 4/74 (7%) were more affected by the UTIs causing bacterial isolates. Besides, 30 (13.4%) and 194 (86.6%) of the patients were detected as symptomatic and asymptomatic, respectively. HIV patients aged between 35 and 44 showed the highest percentage of UTIs (91 (40.6)).

**Table 1:** Socio-demographic and clinical data characteristics of HIV patients (*n* = 224)

Variables	Response	Frequency (n (%))
Gender	Male	74 (33.0)
	Female	150 (67.0)
Residence	Urban	191 (85.3)
	Rural	33 (14.7)
Age	< 24	17 (7.6)
	25–34	41 (18.3)
	35–44	91 (40.6)
	45–54	57 (25.4)
	≥ 55	18 (8.0)
Marital status	Married	89 (39.7)
	Single	43 (19.2)
	Divorced	58 (25.9)
	Windowed	34 (15.2)
Educational status	Tertiary school (> 12)	27 (12.1)
	Secondary school (9–12)	66 (29.5)
	Primary school (1–8)	88 (39.3)
	No formal education	43 (19.2)
Occupation	Employed	63 (28.1)
	Dailey laborer	78 (34.8)
	Housewife	34 (15.2)
	Merchant	41 (18.3)
	Others^a^	8 (3.6)
History of hospitalization	Yes	56 (25.0)
	No	168 (75.0)
History of catheterization	Yes	27 (12.1)
	No	197 (87.9)
IV/oral antibiotic usage in the past 3 months	Yes	75 (33.5)
	No	149 (66.5)
Presence of other chronic disease	Yes	20 (8.9)
	No	204 (91.1)
Recent CD4^+^level	< 200 cells/mm^3^	25 (11.2)
	≥ 200 cells/ mm^3^	199 (88.8)
Recent viral load level	Detected	22 (9.8)
	Not detected	202 (90.2)
ART status	Pre – ART	12 (5.4)
	On –ART	212 (94.6)
ART adherence	Poor	20 (8.9)
	Good	192 (85.7)
History of UTIs	Yes	30 (13.4)
	No	194 (86.6)
Clinical case	Symptomatic	30 (13.4)
	Asymptomatic	194 (86.6)
Fever (> 38 ^0^c)	Yes	27 (12.1)
	No	197 (87.9)
Dysuria	Yes	19 (8.5)
	No	205 (91.5)
Frequency of urination	Yes	26 (11.6)
	No	198 (88.4)
Suprapubic tenderness	Yes	20 (8.9)
	No	204 (91.1)
Urgency of urination	Yes	20 (8.9)
	No	204 (91.1)

*Key*: Others^a^: students and farmers; other chronic diseases: diabetics, cancer.

**Fig. 2:**
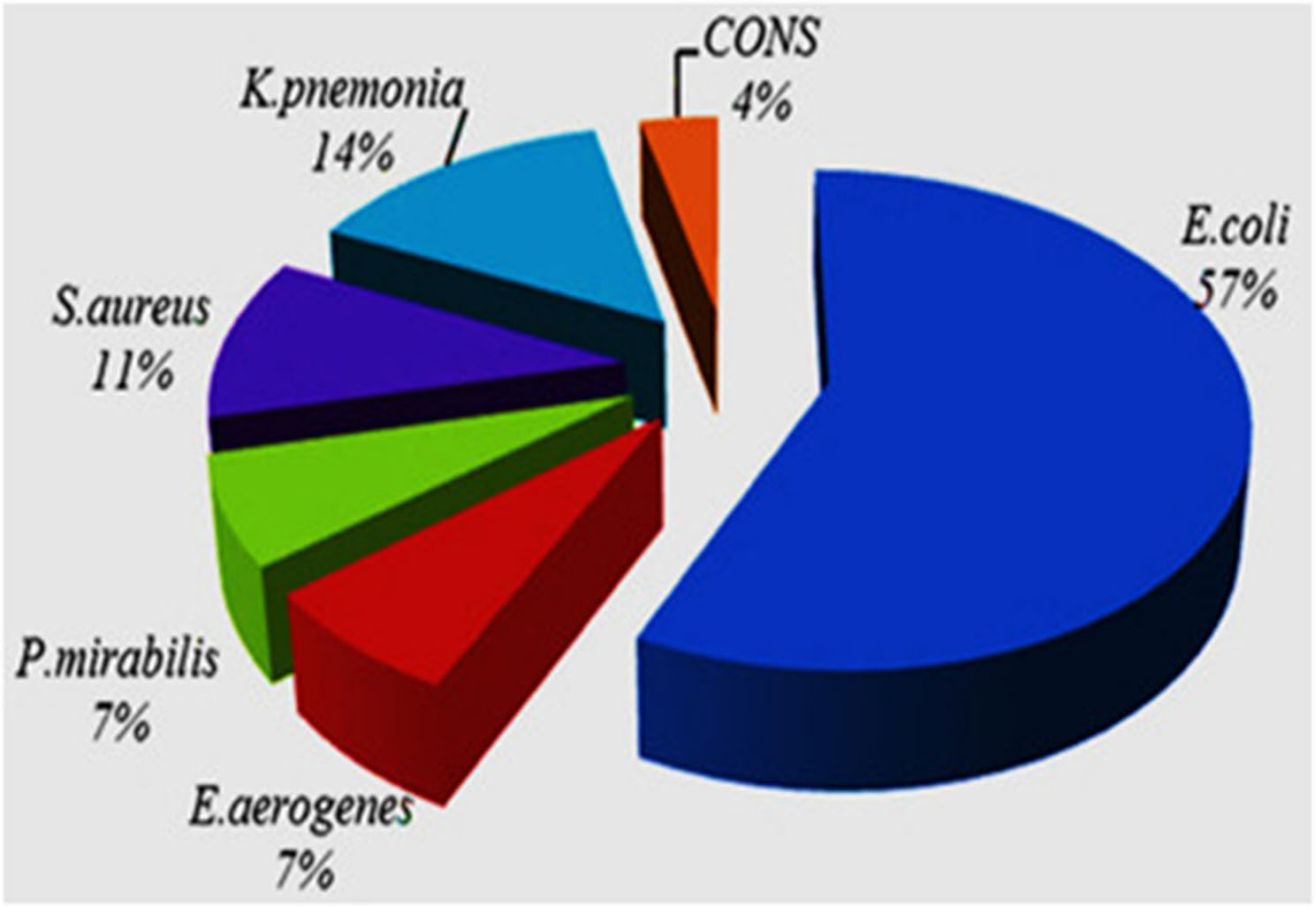
Percentage of UTIs causing bacterial isolates among HIV patients attending

Of the total UTIs causing bacterial isolates, 85% and 25% were Gram-negative and Gram-positive bacteria, respectively (Fig. 2). *E. coli* 16/28 (57%) was the most dominant isolate followed by *K. pneumonia* 4/28 (14%) and *S. aureus* 3/28 (11%). Compared to Gram-positive bacteria, Gram-negative bacteria showed a high percentage in the UTIs causing bacterial isolates.

### Relation of potential factors with UTIs causing bacterial isolates

Among the socio-demographic characteristics, only gender was found to be significantly associated with the growth of UTIs-causing bacteria (Table 2). Following multivariate regression female HIV patients showed significantly at least three times more likely to have been infected by UTIs causing bacteria (AOR = 3.427; 95% CI 1.05, 11.2) as compared to male HIV patients (*P* = 0.042).

**Table 2:** Associated risk factors related to UTIs causing bacterial isolates among HIV patients

Variables		Isolated bacteriuria		COR(95% CI)	P-value	AOR(95% CI)	P-value
		Positive n (%)	Negative n (%)				
Gender	Male	4(5.4)	70 (94.6)	1			
	Female	24 (16)	126 (84)	3.333(1.112–9.995)	0.032	3.427(1.045–11.238)	0.042*
Recent CD4^+^Level	< 200 cells/mm^3^	9 (36)	16(66.7)	5.329(2.074–13.692)	0.001	3.648(1.150-11.574)	0.028*
	≥ 200cells/mm^3^	19(9.5)	180(90.5)	1			
Recent viral load level	Detected	7(31.8)	15(68.2)	4.022(1.473–10.983)	0.007	2.728(0.816–9.125)	0.103
	Not detected	21(10.4)	181(89.6)	1			
History of catheterization	Yes	7(25.9)	20(74.1)	2.933(1.109–7.757)	0.030	1.884(0.580-6.118)	0.292
	No	21(10.7)	176(89.3)	1			
History of UTI	Yes	9 (30)	21(70)	3.947(1.584–9.838)	0.003	3.403(1.212–9.553)	0.020*
	No	19(9.8)	175(90.2)	1			
Presence of other chronic disease	Yes	6 (30)	14(70)	3.545(1.236–10.170)	0.019	2.413(0.739–7.884)	0.145
	No	22(10.8)	182(89.2)	1			
Dysuria	Yes	5(27.8)	13(72.2)	3.060(1.002–9.368)	0.050	0.605(0.125–2.930)	0.532
	No	23(11.2)	183(88.8)	1			
Frequency of urination	Yes	5(19.2)	21(80.8)	1.812(0.623–5.270)	0.275		
	No	23(11.6)	175(88.4)	1			
Suprapubic tenderness	Yes	4 (20)	16(80)	1.875(0.579–6.075)	0.295		
	No	24(11.8)	180(88.2)	1			
Urgency of urination	Yes	4 (20)	16(80)	1.875(0.579–6.075)	0.295		
	No	24(11.8)	180(88.2)	1			

*Key: COR* crude odd ration, *AOR* adjusted odd ratio, *CI* confidence interval, n-number of patients.

The association between the patient’s clinical features and the growth of UTIs-causing bacteria was measured (Table 2). Previous history of UTIs (AOR 3.403; 95% CI 1.2, 9.6) and CD4^+^ count less than 200 cells/mm^3^ (AOR 3.648; 95% CI 1.2, 11.6) were significantly associated with UTIs. However, no significant association was confirmed with having dysuria, other chronic diseases, and previous history of catheterization and UTIs.

### Antimicrobial susceptibility pattern

The susceptibility pattern of Gram-negative and Gram-positive isolates was determined (Table 3). *E. coli* (16), *K. pneumonia* (4), *E. aerogenes* (2), and *P. mirabilis* (2) were resistant to Ampicillin. Whereas all the UTIs-causing bacterial isolates were susceptible to nitrofurantoin. *K. pneumonia* was susceptible to gentamicin, meropenem, and nitrofurantoin, whereas 75% of *K. pneumonia* was susceptible to Ceftriaxone, Norfloxacin Tetracycline, and Ciprofloxacin. *E. coli*, Gram-negative bacteria, the predominant isolate was obtained to be resistant to ampicillin 15 (93.7%), cotrimoxazole 13 (81.3%), and tetracycline 12 (75%). Whereas, sensitive to nitrofurantoin 16 (100%), followed by 15 (93.7%) to ciprofloxacin and gentamicin each, 14 (87.5%) to norfloxacin, chloramphenicol, and meropenem each, and ceftriaxone 12(75%). *P. mirabilis* (2) was to all antibiotics except ampicillin, cotrimoxazole, and tetracycline. *E. arerogenes* was 100% susceptible for meropenem, nitrofurantoin, ceftriaxone, norfloxacin, ciprofloxacin, and chloramphenicol.

**Table 3:** Antibacterial susceptibility pattern of UTIs causing gram-negative bacterial isolates

Bacterial isolate	Pattern	Antibacterial									
		AMP	STX	GN	MER	NIT	CTR	NOR	TET	CIP	CAF
*E. coli* (16)	S	1(6.3)	3 (18.7)	15 (93.7)	14 (87.5)	16 (100)	12 (75)	14 (87.5)	4 (25)	15 (93.7)	14 (87.5)
	R	15 (93.7)	13 (81.3)	1 (6.3)	2 (12.5)	0 (0)	4 (25)	2 (12.5)	12 (75)	1 (6.3)	2 (12.5)
*K. pneumonia* (4)	S	0(0)	1 (25)	4 (100)	4 (100)	4 (100)	3 (75)	3 (75)	3 (75)	3 (75)	2 (50)
	R	4 (100)	3 (75)	0 (0)	0 (0)	0 (0)	1 (25)	1 (25)	1 (25)	1 (25)	2 (50)
*E. aerogenes* (2)	S	0 (0)	1 (50)	1 (50)	2 (100)	2 (100)	2 (100)	2 (100)	1 (50)	2 (100)	2 (100)
	R	2 (100)	1 (50)	1 (50)	0 (0)	0 (0)	0 (0)	0 (0)	1 (50)	0 (0)	0 (0)
*P. mirabilis* (2)	S	0 (0)	0 (0)	2 (100)	2 (100)	2 (100)	2 (100)	2 (100)	0 (0)	2 (100)	2 (100)
	R	2 (100)	2 (100)	0 (0)	0 (0)	0 (0)	0 (0)	0 (0)	2 (100)	0 (0)	0 (0)

*Key*: S: Susceptible, R: Resistant, AMP: ampicillin, CIP: ciprofloxacin, MER: meropenem, CTR: ceftriaxone, NIT: nitrofurantoin, GN: gentamicin, NOR: norfloxacin, TET: tetracycline, CFT: cefoxitin, STX: cotrimoxazole, CAF: chloramphenicol.

Of the UTIs-causing Gram-positive bacteria, *S. aureus* was the dominant bacterium. *S. aureus was* found to be sensitive 3(100%) to cefoxitin, nitrofurantoin, erythromycin, clindamycin, gentamicin, penicillin, and ciprofloxacin (Table 4). Whereas, S. aureus was found to be 100% resistant to cotrimoxazole and ampicillin, and 75% resistant to tetracycline. Of the twelve antibiotics tested, the CONS were resistant only to ampicillin, cotrimoxazole, tetracycline, and ciprofloxacin.

**Table 4:** Antimicrobial susceptibility pattern of UTIs causing Gram-positive bacterial isolates

Bacterial isolates	Pattern	Antibacterials											
		AMP	STX	GN	NIT	CTR	TET	CIP	CAF	PEN	CLN	E	CFT
*S.aureus* (3)	S	0 (0)	0 (0)	3 (100)	3 (100)	2 (75)	1 (15)	3 (100)	2 (75)	2 (75)	3 (100)	3 (100)	3 (100)
	R	3 (100)	3 (100)	0 (0)	0 (0)	1 (25)	2 (75)	0 (0)	1 (25)	1 (25)	0 (0)	0 (0)	0 (0)
*CONS* (1)	S	0 (0)	0 (0)	1 (100)	1 (100)	1 (100)	0 (0)	1 (100)	1 (100)	0 (100)	1 (100)	1 (100)	1 (100)
	R	1 (100)	1 (100)	0 (0)	0 (0)	0 (0)	1 (100)	1 (100)	0 (0)	0 (0)	0 (0)	0 (0)	0 (0)

*Key*: AMP: ampicillin, CIP: ciprofloxacin, NIT: nitrofurantoin, GN: gentamicin, TET: tetracycline, PEN: penicillin, CTR: ceftriaxone, E: erythromycin, CLN: clindamycin, CFT: cefoxitin, STX: cotrimoxazole, CAF: chloramphenicol.

### Multi-drug resistance pattern of the UTIs causing bacterial isolates

The multi-drug resistance (MDR) pattern of the UTIs causing bacterial isolates was evaluated (Table 5). Among the total isolates (*n* = 28), 22(78.6%) of the bacterial isolates were found to be multi-drug resistant (MDR ≥ 2 groups of drugs). All Gram-positive (100%) and 75% of Gram-negative bacterial isolates were resistant to two or more of the drugs that are commonly prescribed in the study area.

**Table 5:** Multi-drug resistance pattern of UTIs causing bacterial isolates

Gram-reaction	Bacterial isolate	Total (n (%))	R1	R2	R3	R4	≥ R5	MDR (n (%))
Gram-negative (24/85%)	*E. coli*	16 (57)	1 (6.3 )	3(18.8 )	4 (25 )	2 (12.5 )	4 (25)	13 (81.3)
	*K. pneumonia*	4 (14)	0 (0)	0(0)	1 (25 )	1 (25 )	1 ( 25)	3 (75)
	*E. aerogenes*	2 (7)	0 (0)	1 (50)	0 (0)	0 (0)	0 (0)	1 (50)
	*P. mirabilis*	2 (7)	0 (0)	0 (0)	0 (0)	1 (50)	0 (0)	1 (50)
Gram positive (4/15%)	*S. aureus*	3 (11)	0(0)	0 (0)	3 (100)	0 (0)	0 (0)	3 (100)
	*CONS*	1 (4)	0(0)	1 (100)	0 (0)	0 (0)	0 (0)	1 (100)
	Total	28 (100)	1(3.6)	5 (17.9)	8 (28.6)	4 (14.3)	5 (17.9)	22 (78.6)

R1; resistance to one group of a drug; R2; resistance to two groups of a drug; R3; resistance to three groups of a drug; R4; resistance to four groups of a drug; R5; resistance to five or more drug; MDR; Multidrug resistance; CONS; coagulase-negative staphylococcus.

## Discussion

The burden of bacterial pathogens that cause UTIs and their resistance to ordinary drugs lines up with immunity depletion among patients with HIV infection [41]. Uropathogens are becoming a public health threat at an alarming rate across the globe; perhaps aggravated in resource-limited settings [42]. Female patients with a previous history of UTIs, and CD4^+^ count < 200/mm^3^ were found to be significantly associated with UTIs-causing bacteria. Whereas current HIV viral load level, history of catheterization, history of other chronic diseases, and dysuria were not significantly associated with females.

In the current study, the overall prevalence of UTIs-causing bacteria isolates among HIV patients was 12.5%. This finding corresponds with previous studies cited from Jimma (12%) [17], Gondar (11.9%) [20], Tanzania (12.3%) [27] and studies conducted elsewhere [43–45]. In contrast, a high prevalence of UTIs causing bacterial isolates among HIV patients was recorded from eastern Ethiopia (18%) [34], Southern Ethiopia (14.1%) [29], studies from India (77.5% and 41.7%) and [46, 47], South Africa (48.7%) [15], Warsaw (23.2%) [28] and other three studies from different states in Nigeria (21.1%, 23.5% and 93.8%) [48–50]. Those huge disparities might be due to the difference in sample size, sample processing techniques, the degree of the immune status of the patients, clinical features of the study patients, ART use, personal and environmental hygiene-related, sexual activity, socio-demographic, and geographical characteristics [13].

Compared to HIV-infected male patients, HIV-infected females who attended ART clinics of ACSH and MGH had about 3 times more chance of developing UTIs. This finding was in line with previous reports from Jimma and Addis Ababa, Ethiopia reported a high prevalence of UTIs causing bacterial isolates among female HIV patients than their male counterparts [17, 31]. Furthermore, a study conducted in Gondar, the northern part of Ethiopia, established a high prevalence of UTIs-causing bacteria among females than males [20]. Evidence from various epidemiological studies showed that UTIs were more common in females than in males [51, 52]. The higher prevalence rate of UTIs among female patients may be due to shorter and wider urethra, lack of prostatic fluid, and moist urethra that favors microbial growth and others are the main reasons for their vulnerability [53]. Additionally, the mechanical introduction of pathogens into the bladder and trauma increases the risk of UTIs among females irrespective of their HIV serostatus [13].

Patients with a previous history of UTIs were found to be three times more likely to develop UTIs compared to those who had never encountered previous UTIs. Previous findings that correspond with this study were reported from Gondar [20], and Addis Ababa, Ethiopia [31]. On the other hand, this result contradicts another study’s findings conducted in Jimma Ethiopia which declares no significant association with current and previous history of UTIs [17]. This might be due to the presence of resistant strains as a result of repeated therapy from those who had a previous history of UTIs and the disparities might be due to adherence to medication and health-seeking behavior differences [13].

UTIs boldly appear in HIV patients as the CD4^+^ level of the patients dropped [54]. In the current study, patients with CD4^+^ count < 200/mm^3^ had a chance to develop UTIs three times higher than their immuno-competent counterparts. This finding was supported by studies conducted in Ethiopia [15, 54] and India [55]. Although this explanation needs further investigation, this could be due to the depressed immunity of the patients implying that as the CD4^+^ counts decline, the risk of UTIs and broadly opportunistic infections also increases [31].

In our study, 85% of UTIs were caused by Gram-negative bacteria. The dominant bacterium isolated in the current study was *E. coli* (57%). Similar studies from Jimma (54.3%) [17], Gondar (56.1%) [20] and Addis Ababa, Ethiopia (49%) [54] reported that *E. coli* was the dominant bacterium to cause UTI. The result of the current finding was also comparable with studies conducted in Nigeria [56] and India [29]. The reason why *E. coli* was found dominant might be due to its most common presence in the vaginal and rectal area [57]. In contrast to this study, other findings from Nigeria and Ghana reported that the dominant bacterium was *S. aureus* with a prevalence rate of 45.33% and 40%, respectively [49, 51]. These variations might be due to sample collection technique and personal and environmental hygiene, and the availability of underlying conditions [13].

More than 80% of Gram-negative bacteria were found to be susceptible to ciprofloxacin, ceftriaxone, gentamycin, nitrofurantoin, chloramphenicol, and norfloxacin. However, most of the Gram-negative bacterial isolates showed resistance to ampicillin, cotrimoxazole, and tetracycline. The finding was similar to studies reported from other areas [17, 31]. Due to their distinctive structure, Gram-negative bacteria are more resistant than Gram-positive bacteria. Most antibiotics must pass the outer membrane to access their targets, for example, hydrophobic drugs can pass through by a diffusion pathway, on the other hand, hydrophilic antibiotics like β-lactams pass through porins, and vancomycin can’t cross the outer membrane due to their structure that hinder it from using any of these passages. Any alteration in the outer membrane by Gram-negative bacteria like changing the hydrophobic properties or mutations in porins and other factors can create resistance.

Gram-positive bacteria lack this important layer, which makes Gram-negative bacteria more resistant to antibiotics. Decreasing outer membrane permeability of Gram-negative bacteria is the main reason for resistance to a wide range of antibiotics [58]. Higher resistance rates of these bacteria could be considered as great threats and alarm the stakeholders to have more surveillance and control of the use of antimicrobials to combat infection [59].

From the Gram-positive isolates, most of the *S. aureus* isolates showed high-level of susceptibility to ciprofloxacin, nitrofurantoin, gentamicin, penicillin, ceftriaxone, erythromycin, clindamycin, and cefoxitin. On the other hand, most of the *S. aureus* isolates were resistant to ampicillin, cotrimoxazole, and tetracycline which was corroborative with other studies conducted in different parts of Ethiopia [13, 31]. Our result was similar with Tanzanian study revealed that Gram-positive bacteria were the most prevalent isolates with high sensitivity to nitrofurantoin, followed by gentamycin [60]. In Uganda, *Staphylococcus aureus* showed sensitivity to ciprofloxacin, nitrofurantoin, and gentamycin [5, 6]. In Nigeria, there is a growing concern over resistance to various antibiotics, including ampicillin, tetracycline, chloramphenicol, co-trimoxazole, gentamicin, augmentin, vancomycin, cefuroxime, nitrofurantoin, and ofloxacin [61] .

Generally, most of the bacterial isolates were susceptible to ciprofloxacin, ceftriaxone, gentamicin, nitrofurantoin, and meropenem. Whereas the bacterial isolates were very resistant to ampicillin, cotrimoxazole, and tetracycline. These findings were in agreement with the previous finding from Ethiopia [31], and South Africa [62]. This might be due to differences in the wide prescription of the drugs or the fact that the common use of cotrimoxazole is prophylaxis against HIV-associated opportunistic infections.

Antimicrobial resistance is a major clinical problem in treating infections caused by different bacterial pathogens and has increased dramatically over the current years. Multidrug resistance, which has countless implications on the health outcome of HIV patients, was observed in our study. In the current study, 78.6% of the bacteria isolates were multidrug resistant. This was higher compared to the finding reported in Mysore, India (58.3%) [63]. But, it was lower than the reports obtained from Gondar, Ethiopia (95%) [20], and Port Harcourt in Nigeria (92.8%) [64]. The antibiotic resistance pattern observed in our study could be due to antibiotic abuse, circulation of high fake drugs, use of antibiotics for animal farming, self-medication, low cost, and inappropriate use of antimicrobial agents by patients and practitioners [65].

### Limitations of the study

The cross-sectional nature of our study design is the primary limitation due to a lack of testing facilities and the unprecedented genocidal war waged by the government of Ethiopia and its allies on the Tigray people, in Northern Ethiopia. We did not attempt to identify other causative agents like anaerobic UTIs causing bacteria that would have made a significant contribution to a true prevalence of UTIs causing bacteria in HIV patients. Although the current study is important in terms of identifying UTIs causing bacteria and determining their antimicrobial sensitivity that provides precise scientific data for appropriate treatment, prevention, and control of UTIs, we believe that these data are not sufficient to know the magnitude of all UTIs causing bacteria. Moreover, to identify and evaluate the isolates in terms of drug-resistant and virulence factor genes molecular studies shall have been done but it was not done.

## Conclusions

In the current study, the overall prevalence of UTIs causing bacterial isolates among people living with HIV was 12.5%. Factors such as sex, CD4^+^ count < 200 cells/mm^3,^ and previous history of UTIs were significantly associated with the prevalence of the UTIs causing bacterial isolates. Of the bacterial isolates, *E. coli* was found to be the most predominant bacteria. Most of the bacterial isolates were susceptible to ciprofloxacin, ceftriaxone, gentamicin, nitrofurantoin, and norfloxacin but, resistant to ampicillin, cotrimoxazole, and tetracycline. As the antibiotic susceptibility pattern of UTIs causing bacteria to various antibiotics varies, management of UTIs among HIV-infected individuals is needed. To bring immunological and virological recovery, HIV patients shall get enough awareness and emphasis on care for female HIV patients through the setting of different intervention modalities. A molecular study is mandatory to identify genes responsible for drug resistance and virulence process.

### Electronic supplementary material

Below is the link to the electronic supplementary material.

Supplementary Material 1

## Data Availability

The datasets generated and/or analyzed during the current study are not publicly available because of the sensitive nature of the data but are accessible from the corresponding author at a reasonable request.

## Abbreviations

ART: Antiretroviral therapy
CD4: Cluster of differentiation 4
CONS: Coagulase negative staphylococcus
HIV: Human Immunodeficiency Virus
MDR: Multidrug resistance
UTI: Urinary tract infection
WHO: World Health Organization
ACSH: Ayder Comprehensive Specialized Hospital
MGH: Mekelle General Hospital
