# Supplementary material for: Antimicrobial susceptibility patterns of urinary tract infections causing bacterial isolates and associated risk factors among HIV patients in Tigray, Northern Ethiopia
Source: BMC Microbiol. 2024 Apr 27;24:148. doi: 10.1186/s12866-024-03297-2 (PMC11055276; doi:10.1186/s12866-024-03297-2)
Supplement: Supplementary file 1 — Supplementary Material 1 [file 12866_2024_3297_MOESM1_ESM.docx]

**1: Questionnaire/ English Version**

Questionnaire for Socio-demographic and clinical investigation for bacteriuria isolates causing UTI among HIV patients attending Ayder comprehensive specialized hospital and Mekelle Hospital ,Mekelle , Northern Ethiopia. I request kindly to give appropriate response for each question. Your response will be kept confidential.

Date _________/______/______

S.No __________________

**I. Socio-demographic** **Variables**

1. Gender? A. Male B. Female
2. Age in years? ___________________
3. Where is your Place of residence?

A. Urban B. Rural

4. What is your marital status?

A. Single C. Divorced

B. Married D. Windowed

5. What is your educational status?

A.No formal education C. Secondary school

B. Primary school D. Collage and above

E. Other (specify)______________

6. What is your occupation status?

A. Unemployed D. Farmer

B. Employee E.Housewife

C. Student F.Others (specify)______________

**II. Clinical Data**

8. Have you ever admitted to any health facility?

A. Yes B. No

9. Have you ever used catheter during your health facility stay?

A. Yes B. No

10. Have you taken intravenous or oral antibiotic usage in the past 3 month?

A. Yes B.No

11. Presence of other chronic disease?

A. Yes B. No

12. Most recent CD4 count?___________________

13. Most recent viral load level?_________________

14. ART treatment?

A. Pre-ART B. On ART

15. ART Adherence?

A. Good B. Poor

16. Have you ever seen previous history of UTI?

A. Yes B. No

17. Clinical case?

A.Symptomatic B. Asymptomatic

18. Do you have current symptoms of UTI? if Symptomatic**?**

Yes No

A**.** Fever (T >38^0^C)

B. Urgency

C.Frequency

D.Dysuria

E.Suprapubic tenderness

F. Other (specify) __________________

Collected by: Name _____________________________Sign__________

Checked by Supervisor: Name ______________________Sign__________

**Annex XIII: Laboratory data recording format**

Code No __________________

1. Date and time of specimen collection______________
2. Specimen appearance ________________
3. Specimen type____________________
4. Urine Microscopy

E. Gram stain Result of urine ________________

F. Cultures and Identification

1. Colony count

- <10^3^CFU/ml
- 10^3^CFU/ml
- 10^4^CFU/ml
- >10^5^ CFU/ml

1. Significant bacteruria:

- Yes
- No

1. Growth characteristics

Blood agar

- Alpha Hemolysis
- Beta hemolysis
- No hemolysis

MacConkey agar

- Lactose fermenter
- Non Lactose fermenter
